# Quantifying genetic load through joint modelling of inbreeding depression and inbreeding load for litter size in rabbits

**DOI:** 10.1186/s12711-026-01090-5

**Published:** 2026-09-20

**Authors:** C. Hervás-Rivero, A. T. Nguyen, G. Kövér, A. Bokor, I. Nagy, I. Curik, L. Varona

**Affiliations:** 1 Departamento de Anatomía, Embriología y Genética Animal, Universidad de Zaragoza, Instituto Agroalimentario de Aragón (IA2), Zaragoza, Spain; 2 Hungarian University of Agriculture and Life Sciences - Institute of Animal Sciences (MATE), Kaposvár, Hungary; 3 University of Zagreb-Faculty of Agriculture, Zagreb, Croatia

## Abstract

**Background:**

Genetic load, defined as the burden of deleterious variants carried by a population, has two components: a realized one that is expressed as reduced fitness, and a hidden one that is masked in heterozygotes. The hidden component, arising from recessive (or partially recessive) deleterious alleles and from overdominant loci, constitutes the inbreeding load (IL), which is unmasked under inbreeding and observed as inbreeding depression. Conventional analyses focus on average inbreeding depression and assume homogeneous effects across ancestors. In contrast, the IL framework allows heterogeneous ancestral contributions to be modelled through Mendelian decomposition. For composite reproductive traits, such as number born alive (NBA) in rabbits, inbreeding may act through distinct biological pathways associated with both the dam and the litter. We developed and validated an integrated model that jointly partitions maternal and litter IL underlying NBA in rabbits.

**Results:**

Data consisted of 33,521 kindling records from a Pannon White rabbit population and a pedigree of 43,858 individuals. Total inbreeding was decomposed into partial ancestral contributions using a tabular Mendelian decomposition, allowing IL effects to be modelled as additive genetic random effects. Bayesian linear mixed models varying in maternal and litter IL effects, inbreeding depression covariates, and genetic correlations genetic correlations between the additive effect and each of the two IL measures, as well as between the two IL measures, were compared by the Deviance Information Criterion; the model with both IL effects but no genetic correlations fitted best. Average inbreeding depression was consistently negative, with litter inbreeding showing a stronger and more precisely estimated effects on NBA than maternal inbreeding. Both maternal and litter IL variances were substantial, indicating marked heterogeneity among ancestors in their contributions to inbreeding depression. Analysis of genetic trends revealed sustained genetic progress for NBA and litter IL, accompanied by opposite temporal trends for maternal IL.

**Conclusions:**

Joint modelling of maternal and litter IL provides a deeper and biologically coherent understanding of the genetic architecture of litter size in rabbits. This approach reveals hidden tensions between selection for production and biological fitness, highlighting the need for breeding strategies that balance additive genetic progress with the management of hidden genetic load.

**Supplementary Information:**

The online version contains supplementary material available at https://doi.org/10.1186/s12711-026-01090-5.

## Background

Inbreeding results from the mating of related individuals and is an unavoidable consequence of selection in finite populations. Inbreeding affects both the mean and variance of economically relevant traits [1]. The most common consequence of inbreeding is inbreeding depression (ID), defined as the decline of the phenotypic performance of reproductive and fitness-related traits with inbreeding [2, 3]. This decline primarily arises from two mechanisms, the loss of heterozygote advantage due to overdominance or partial dominance, and the increased expression of deleterious recessive alleles, defined as alleles that have detrimental effects on fitness or other phenotypic traits, in homozygous state.

Genetic load is broadly understood as the burden of deleterious genetic variants carried by individuals in a population, which reduces mean fitness when expressed, regardless of mode of gene action [4]. Genetic load comprises a realized component that is expressed in the population through reduced phenotypic performance, and a hidden component, which is carried in heterozygous form. This hidden component - arising from recessive or partially recessive deleterious alleles, and from overdominant loci, that are concealed in the heterozygous state - constitutes the inbreeding load (IL). When increased homozygosity exposes IL through inbreeding, its realized expression is observed as inbreeding depression [5]. More specifically, IL reflects the potential of ancestors to generate inbreeding depression in their inbred descendants.

Traditionally, ID has been estimated by regressing phenotypic records on the inbreeding coefficient (b_F_), without distinguishing heterogeneous contributions of individual ancestors to deleterious variation [6]. To overcome this limitation, Casellas [7] proposed the concept of individual inbreeding load (IL), defined as the cumulative effect of deleterious alleles that an individual can transmit to its inbred progeny. Although IL is not expressed by the carrier itself, its effects only become evident in its inbred descendants.

Unlike classical analyses, the IL is defined as the expected combined impact of all inbreeding-related polymorphisms in a hypothetical 100% inbred descendant from each specific ancestor [7, 8]. Estimation of IL requires Mendelian decomposition of inbreeding [9] by partitioning an individual’s total inbreeding into partial contributions attributable to specific ancestors. These partial inbreeding coefficients can then be included in linear mixed models [8] to predict IL as an additional additive genetic effect. Consequently, all individuals have two additive genetic effects, the direct additive genetic effect, which is expressed in its own phenotype, and the additive IL effect, which is expressed only in its inbred progeny. Moreover, these two additive genetic effects may be genetically correlated [8]. This methodology has already been applied across several species and traits, including beef cattle growth [8], dairy cattle fertility [10], milk yield in dairy sheep [11], and equine reproductive traits [12].

In rabbit production, number born alive (NBA) is one of the most economically relevant reproductive traits because of its direct impact on farm productivity and profitability [13]. ID is known to reduce NBA through decreased ovulation rate, increased embryonic mortality, and reduced kit survival at birth [14–16]. Therefore, both the inbreeding level of the dam and that of the kits are expected to influence phenotypic performance. To disentangle these pathways, it is essential to consider the functional relationships between the biological components underlying litter size. As extensively described by Baselga and Blasco [17], NBA is a composite trait determined by the ovulation rate and prenatal survival, which can be further partitioned into pre-implantation and post-implantation survival. Their work established that, although ovulation rate defines the physiological upper limit of litter size, uterine capacity and embryonic survival constitute the primary limiting factors [17, 18]. Consequently, the accumulation of inbreeding may differentially disrupt these biological components, impairing either the dam’s ability to release oocytes or the genetic potential of the embryos to survive gestation. Thus, NBA may be influenced by multiple IL pathways, as both the dam and the kits inherit independent IL from their ancestors. The aim of this study was to develop and validate a unified modelling framework that simultaneously incorporates maternal and litter inbreeding loads, enabling the dissection of genetic load into distinct ID pathways for a composite reproductive trait such as number born alive (NBA) in a rabbit population.

## Materials and methods

### Dataset

The dataset consisted of 33,521 kindling records (recorded between 1992 and 2024) from the Pannon White rabbit breed, which was developed at Kaposvár University in the late 1980s and officially recognized in Hungary in 1992 [19]. The average number of kits born alive per kindling was 9.02 (± 3.43). The pedigree was recorded between 1989 and 2024 and it included 43,858 109 individuals, of which 34,761 (79.2%) were inbred, with an average inbreeding coefficient of 0.057 110 (± 0.052). For individuals with available phenotypic records, the average pedigree depth was 18 generations. The rabbit does were kept in a closed rabbitry and were caged individually. They were fed a commercial pelleted diet, and water was provided through nipple drinkers. The breed has been selected for litter weight at day 21 and average daily gain.

### Mendelian decomposition of inbreeding

First, we decomposed total inbreeding into partial inbreeding coefficients attributable to each ancestor using the algorithm of Garcia-Cortés et al. [9]. This approach accounts for the Mendelian sampling associated with each ancestor and is based on Malécot’s tabular method [20], with specific modifications, as follows:

1 $$\:{{\Phi\:}}_{ij}=\frac{1}{2}\left({{\Phi\:}}_{is'}+\:{{\Phi\:}}_{id'}\right)+\:{\delta\:}_{ij},$$

where Φ is the coancestry or kinship [20] for individuals *i* and *j*; and *s*′ and *d*′ are the parents of animal j. In the above, an additional term, *δ*_*ij*_, referred to as Mendelian sampling and associated with intrafamilial variation, is incorporated. The diagonal elements *δii* are calculated as follows:

(1) when both parents are known,

2 $$\:{\delta\:}_{ii}=\frac{1}{8}\left(1-\:{f}_{s}\right)+\frac{1}{8}\left(1-\:{f}_{d}\right),$$

(2) when only one parent is known, and

3 $$\:{\delta\:}_{ii}=\frac{1}{4}+\frac{1}{8}\left(1-\:{f}_{k}\right),$$

(3) when none of them are known

4 $$\:{\delta\:}_{ii}=\frac{1}{2}\:,$$

where *f*_*s*_ refers to the inbreeding coefficient of the sire, *f*_*d*_ to the inbreeding coefficient of the dam (doe), and *f*_*k*_ to the inbreeding coefficient of the known parent (used when only one parent is known).

Thus, the tabular method was applied starting from the *δ* value of each individual, by verifying the kinship between all possible pairs. The Mendelian decomposition of an individual’s inbreeding with respect to each ancestor corresponds to the kinship of its parents, as represented in the parental tabular matrix. This decomposition enables the inbreeding load to be modelled as an additive trait [7]. These calculations were implemented using a custom program written in Fortran90, available under request.

### Models for analysis

The model of analysis of NBA (model 1) was:

Model 1 $$\begin{aligned}\mathbf{y =} &\mathbf{f_{doe}b_{fdoe}+ f_{lit}b_{flit}} \cr&\mathbf{+ Sh +Wp +Xd + Za} \cr&\mathbf{+ Z_{doe}K_{idoe} + Z_{lit}K_{ilit} + e} \end{aligned} $$

where 𝐲 is the vector of phenotypic records, ***b***_***fdoe***_ and ***b***_***flit***_ are the regression coefficients representing inbreeding depression per unit of inbreeding, and represent the average ID of does and litters, respectively, **d** is the vector of fixed effects (including order of parity with 6 levels), **h** is the vector of random herd–year–season (318 levels), **p** is the vector of the permanent environmental maternal effects, **a** is the vector of the direct additive genetic effects, 𝐢_**doe**_ is the vector of the IL effects associated to doe inbreeding, 𝐢_**lit**_ is the vector of the IL effects associated with inbreeding of the kits, and ***e*** stands for the vector of residuals. Moreover, **f**_**doe**_ and **f**_**lit**_ are the vectors of total inbreeding coefficients from the dam and the litter respectively, and **X**,** S**,** Z**, **W**,** Z**_**doe**_, and **Z**_**lit**_ are the associated incidence matrices. The **K** matrix is lower triangular, **K** = **T(I-P)**, where **T** contains the partial inbreeding coefficients associated with all individuals or litters, **I** is the identity matrix, and matrix **P** has 0 on its diagonal and 0.5 for elements that connect an individual with its sire and dam. To reduce the computational burden caused by very low coefficients, only the elements of **K** with absolute values greater than 0.001 were retained.

To illustrate the impact of using alternative pathways for the calculation of IL, a simplified inbred pedigree is presented in Appendix A. This example compares the doe-based and litter-based pathways using the same individuals and phenotypic records, highlighting how the Mendelian decomposition differs between approaches. These differences generate distinct allocations of partial inbreeding coefficients across records and, consequently, lead to different covariates entering the mixed model equations.

The assumed variances and covariances among random effects are:

5 $$\:var\left(\begin{array}{c}a\\\:{i}_{doe}\\\:{i}_{lit}\\\:p\\\:e\end{array}\right)=\:\left[\begin{array}{ccccc}\mathbf{A}{\sigma}_{a}^{2}&\:\mathbf{A}{\sigma}_{a,idoe}&\:\mathbf{A}{\sigma}_{a,ilit}&\:0&\:0\\\:{\mathbf{A}\sigma}_{a,idoe}&\:\mathbf{A}{\sigma}_{idoe}^{2}&\:{\mathbf{A}\sigma}_{idoe,ilit}&\:0&\:0\\\:{\mathbf{A}\sigma}_{a,ilit}&\:{\mathbf{A}\sigma}_{idoe,ilit}&\:\mathbf{A}{\sigma}_{ilit}^{2}&\:0&\:0\\\:0&\:0&\:0&\:\mathbf{I}{\sigma}_{p}^{2}&\:0\\\:0&\:0&\:0&\:0&\:\mathbf{I}{\sigma}_{e}^{2}\end{array}\right]$$

Here, 𝜎_a, idoe_ is the covariance between the additive genetic and the IL effects associated with the mother, $\:{\sigma\:}_{a,ilit}$ is the covariance between the additive genetic and the IL effects associated with the litter, $\:{\sigma\:}_{idoe,ilit}$ is the covariance between the IL effects associated with the mother and the IL effects associated with the litter, $\:{\sigma\:}_{a}^{2}$ is the additive genetic variance, $\:{\sigma\:}_{idoe}^{2}$ the variance of the IL effects associated with the mother, $\:{\sigma\:}_{ilit}^{2}$ the variance of the IL effects associated with the litter, $\:{\sigma\:}_{p}^{2}$ is the variance of the permanent effect, and $\:{\sigma\:}_{e}^{2}$ is the residual variance. A reduced version of this model (model 0) was also fitted, with the assumption of zero genetic correlations between the direct and IL genetic effects, as well as between the two IL effects to discard potential over–parametrisation.

We also proposed eight additional reduced models. Models 2 and 3 have only one of the IL effects but both inbreeding coefficients:

Model 2 $$\begin{aligned} \mathbf{y =}&\mathbf{ f_{doe}b_{fdoe}+ f_{lit}b_{flit}+ Sh} \cr&\mathbf{+Wp +Xd + Za} \cr&\mathbf{+ Z_{doe}Ki_{doe} + e} \end{aligned} $$

Model 3 $$\begin{aligned} \mathbf{y =}& \mathbf{f_{doe}b_{fdoe}+ f_{lit}b_{flit}+ Sh}\cr& \mathbf{+Wp +Xd + Za} \cr&\mathbf{+ Z_{lit}Ki_{lit} + e}\end{aligned} $$

Models 4 and 5 only have one average depression and one IL effect (doe or dam):

Model 4 $$\begin{aligned} \mathbf{y =}& \mathbf{ f_{doe}b_{fdoe}+ Sh} \cr& \mathbf{+Wp +Xd + Za} \cr& \mathbf{+ Z_{doe}Ki_{doe} + e}\end{aligned} $$

Model 5 $$\begin{aligned} \mathbf{y =}& \mathbf{f_{lit}b_{flit}+ Sh} \cr&\mathbf{+Wp +Xd + Za} \cr&\mathbf{+ Z_{lit}Ki_{lit} + e} \end{aligned} $$

The four remaining models did not account for IL effects: model 6 considered both doe and litter average inbreeding, models 7 and 8 accounted for only one of these effects, and the simplest model 9, excluded inbreeding information altogether.

Model 6 $$\begin{aligned} \mathbf{y =}& \mathbf{f_{doe}b_{fdoe}+ f_{lit}b_{flit}}\cr&\mathbf{+ Sh +Wp +Xd} \cr&\mathbf{+ Za + e} \end{aligned} $$

Model 7 $$\begin{aligned} \mathbf{y =}&\mathbf {f_{doe}b_{fdoe}+ Sh +Wp}\cr& \mathbf{+Xd + Za + e}\end{aligned} $$

Model 8 $$\begin{aligned} \mathbf{y =}& \mathbf{f_{lit}b_{flit}+ Sh +Wp} \cr&\mathbf{+Xd + Za + e} \end{aligned} $$

Model 9 $$\begin{aligned} \mathbf{y =} \mathbf{Sh +Wp +Xd + Za + e} \end{aligned} $$

All models that include **i**, also included genetic correlations between additive genetic and additive IL effects. In model 1, the genetic correlation between **i**_**doe**_ and **i**_**lit**_ was also estimated. As previously mentioned, to discard over-parametrisation, model 1 was also used under the assumption of null genetic correlations (model 0).

It should be noted that, in the proposed models, litters contribute to the phenotypic records only through inbreeding effects and not via their own additive genetic effects. In principle, an alternative set of models could include the additive genetic effects of the kits. However, complementary analyses presented in Additional file 1, Table S1 show that their contribution is negligible and, therefore, they were not included in the present study.

All models were implemented using a Bayesian analysis through a Gibbs sampling approach. The prior distribution of the systematic effects was assumed to be flat, and the prior distribution of the variances components was assumed to be weakly informative with one degree of freedom. The Gibbs sampler was implemented with custom fortran90 software. For each model, we implemented a single long chain of 10,000,000 samples, and a burn-in period of 4,000,000 samples. Thinning was set to every 10th sample to reduce autocorrelations between consecutive draws in order to avoid storing a very large number of samples. Convergence of the Gibbs sampler was assessed using the Geweke test [21]. Finally, model fit was compared using the Deviance Information Criterion (DIC) [22].

## Results

### Inbreeding and inbreeding decomposition

For a total of 33,521 kindling records, 7594 dams were included in the pedigree. Among them, 1499 dams (19.7%) were non-inbred, 3334 (49.3%) had an inbreeding coefficient greater than 0.05, 1701 (22.3%) greater than 0.10, and only 16 (0.2%) had an inbreeding coefficient over 0.25. The average inbreeding coefficient of dams was 0.05 (± 0.04 SD). For litters, 6821 (20.3%) were non-inbred, 15,418 (45.9%) had inbreeding coefficients above 0.05, 8489 (25.3%) above 0.10, and 145 (0.4%) above 0.25. A graphical representation of the yearly evolution of litter and doe inbreeding coefficients is provided in Additional file 2 Fig. S1.

The Mendelian decomposition of the pedigree produced 25,826,446 coefficients derived from only 3236 individuals. The average partial inbreeding coefficient was 9.69 × 10⁻⁵ (± 6.15 × 10⁻⁴ standard deviations; SD). Most of these partial inbreeding coefficients contributed less than 0.001 to descendant inbreeding and were excluded from further analyses. After excluding negligible contributions, we used 152,687 partial inbreeding coefficients for the decomposition of litter inbreeding, and 150,746 for the decomposition of dam inbreeding.

### Model comparison

Posterior estimates of the additive variance, random herd-year-season variance, permanent environmental maternal variance, and the residual variance components and the results of the model comparison with the Deviance Information Criterion (DIC) are presented in Table 1.

**Table 1:** Model comparison (ΔDIC) and posterior means with 95% highest posterior density intervals (HPD95%) of variance components that were shared between the ten fitted models

Model	σ^2^_a_ [HPD95%]	σ^2^_h_ [HPD95%]	σ^2^_*p*_ [HPD95%]	σ^2^_e_ [HPD95%]	ΔDIC
0	0.56 [0.40, 0.73]	0.25 [0.19, 0.32]	0.98 [0.82, 1.15]	9.80 [9.64, 9.97]	0
1	0.56 [0.40, 0.73]	0.25 [0.19, 0.32]	0.99 [0.82, 1.15]	9.80 [9.64, 9.97]	25
2	0.57 [0.41, 0.74]	0.25 [0.19, 0.31]	0.99 [0.83, 1.16]	9.81 [9.64, 9.97]	66
3	0.56 [0.40, 0.72]	0.25 [0.19, 0.32]	0.99 [0.82, 1.15]	9.80 [9.64, 9.97]	27
4	0.53 [0.36, 0.67]	0.25 [0.19, 0.32]	0.99 [0.87, 1.20]	9.82 [9.64, 9.97]	202
5	0.51 [0.37, 0.61]	0.25 [0.19, 0.31]	0.99 [0.84, 1.18]	9.81 [9.66, 9.99]	39
6	0.56 [0.40, 0.73]	0.25 [0.19, 0.32]	0.99 [0.83, 1.16]	9.81 [9.64, 9.98]	71
7	0.52 [0.36, 0.68]	0.25 [0.19, 0.32]	1.03 [0.87, 1.20]	9.81 [9.64, 9.98]	69
8	0.52 [0.36, 0.69]	0.25 [0.19, 0.32]	1.02 [0.85, 1.19]	9.82 [9.66, 9.99]	219
9	0.48 [0.33, 0.65]	0.25 [0.19, 0.32]	1.05 [0.89, 1.22]	9.82 [9.66, 9.99]	257

σ^2^_a_ additive variance; σ^2^_h_ random herd-year-season variance; σ^2^_p_ permanent variance; σ^2^_e_ residual variance; difference in Deviance Information Criterion (DIC) for each model, calculated relative to the model with the best fit (Model 0)

Model 0 (Table 1) provided the best fit to the data, with a DIC value of 723,909. Estimates of the shared variance components were highly consistent across models (Table 2). Posterior means of additive genetic variance (σ²ₐ) ranged narrowly from 0.48 to 0.57, while the corresponding random herd-year-season variance (σ²ₕ) estimates remained constant at 0.25 across all models. Permanent variance (σ²ₚ) estimates showed only minimal variation (0.98–1.05), and residual variance (σ²ₑ) estimates were remarkably stable, with values around 9.8.

Posterior estimates and HPD95% for litter and doe ID and the difference between them are presented in Table 2.

**Table 2:** Posterior means and 95% highest posterior density intervals (HPD95%) of litter and doe inbreeding depression (ID) estimates across alternative models

Model	b_Flit_ [HPD95%]	b_Flit_ – scaled [HPD95%]	b_Fdoe_ [HPD95%]	b_Fdoe_ – scaled [HPD95%]		Δb_F_ [HPD95%]	% (b_Fdoe_> b_Flit_)
0	-5.17 [-6.89, -3.48]	-0.57 [-0.76, -0.38]	-2.99 [-5.48, -0.49]	-0.33 [-0.60, -0.05]	-2.18 [-5.03, 0.96]		91.5
1	-5.60 [-7.49, -3.75]	-0.62 [-0.83, -0.41]	-2.30 [-4.92, 0.37]	-0.25 [-0.54, 0.04]	-3.30 [-6.92, 0.18]		97.2
2	-4.96 [-6.45, -3.47]	-0.54 [-0.71, -0.38]	-2.63 [-5.36, -0.01]	-0.29 [-0.59, -0.00]	-2.33 [-4.52, 1.06]		88.9
3	-5.33 [-7.20, -3.50]	-0.59 [-0.80, -0.39]	-3.59 [-5.67, -1.52]	-0.39 [-0.63, -0.17]	-1.74 [-5.50, 0.90]		91.8
4			-3.67 [-6.24, -1.19]	-0.40 [-0.69, -0.13]			
5	-5.39 [-7.19, -3.64]	-0.59 [-0.80, -0.40]					
6	-4.89 [-6.38, -3.40]	-0.54 [-0.71, -0.37]	-3.57 [-5.63, -1.49]	-0.39 [-0.62, -0.16]	-1.32 [-3.95, 1.31]		83.8
7	-5.08 [-6.57, -3.60]	-0.56 [-0.72, -0.40]					
8			-4.07 [-6.12, -2.02]	-0.45 [-0.68, -0.22]			

b_Flit_ estimated ID in absolute values (kits per litter) of the litter, b_Fdoe_ estimated ID in absolute values (kits per litter) of the doe, Scaled b_Flit_ and b_Fdoe_ are the estimated ID scaled on the mean of the trait for litter and doe respectively, Δb_F_ is the difference between b_Flit_ - b_Fdoe_, % (b_Fdoe_> b_Flit_) percentage of Gibbs samples where b_Fdoe_ was higher than b_Fdoe_

Posterior distributions of the ID parameters provided consistent evidence for a negative effect of inbreeding, with particularly strong support for the litter effect (Table 2). Estimates of litter ID (b_F_ₗ_i_ₜ) were highly stable across models, with posterior means ranging from − 4.89 to -5.60. In all models for which b_F_ₗ_i_ₜ was included, its HPD95% intervals did not included zero, indicating a robust and well-supported detrimental effect of litter inbreeding on the trait. Estimates of doe ID (b_Fdoe_) were likewise negative across models, with posterior means between − 2.30 and − 3.59. The associated HPD95% intervals were generally wider than those for b_F_ₗ_i_ₜ, reflecting lower precision of the maternal effect estimates.

Notably, the HPD95% interval of maternal ID included zero only in model 1, whereas all other models supported a negative maternal ID. This indicates that, while the direction of the b_Fdoe_ effect is consistently negative, its magnitude is more sensitive to model specification. Finally, the posterior mean estimates of the difference between b_F_ₗ_i_ₜ and b_Fdoe_ were consistently negative, with a posterior probability of being lower than zero, measured as the percentage of negative Gibbs samples, ranging from 83.8 (model 6) to 97.2% (model 1).

Results from traditional regression approaches of models 6, 7, and 8 are consistent with those obtained for model 0 (reduced model), supporting the robustness of the estimates across model specifications. In particular, the posterior mean of b_F_ₗ_i_ₜ was highly stable across models, with values for models 6 (-4.89) and 7 (-5.08) being very close to that of model 0 (-5.17), and with largely overlapping HPD95% intervals. In contrast, the estimate of b_Fdoe_ showed greater variability, with model 6 (-3.57) and especially model 8 (-4.07) yielding more negative values than model 0 (-2.99), although their HPD95% intervals still overlapped.

**Fig. 1:**
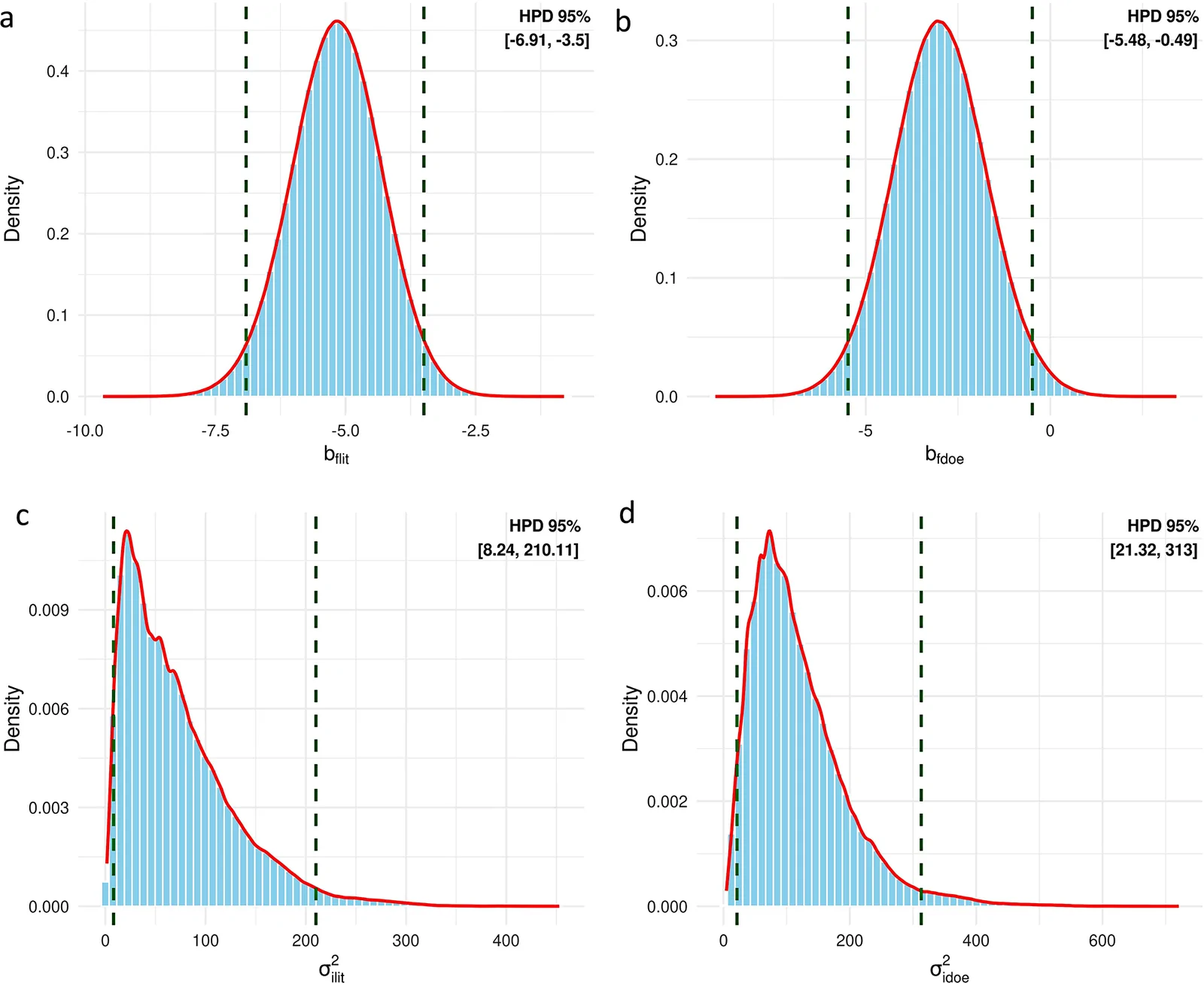
Posterior distributions of inbreeding depression effects. Posterior distributions of inbreeding depression effects (**a**) litter, (**b**) doe and inbreeding load variance (**c**) litter, (**d**) doe for model 0. Green dashed vertical lines indicate the 95% highest posterior density intervals, with corresponding values reported in the upper-right corner of each panel

**Table 3:** Posterior means and 95% highest posterior density intervals (95%HPD) of estimation of litter and doe inbreeding load (IL) related components of variance and genetic correlations across models

Model	σ^2^_ilit_	σ^2^_idoe_	*r*(a, i_lit_)	*r*(a, i_doe_)	*r*(i_lit_,i_doe_)
0	72.24 [2.22, 179.99]	118.47 [6.83, 261.55]			
1	61.07 [1.11, 176.31]	88.66 [2.80, 256.76]	0.39 [-0.36, 0.96]	-0. 42 [-0.95, 0.33]	-0.53 [-0.99, 0.50]
2		76.41 [7.76, 180.88]		-0. 36 [-0.95, 0.73]	
3	44.89 [1.24, 116.29]		0.34 [-0.65, 0.95]		
4		66.44 [4.55, 161.81]		-0.21 [-0.94, 0.85]	
5	45.02 [1.24, 116.29]		0.24 [-0.72, 0.94]		

σ^2^_ilit_ litter IL variance, σ^2^_idoe_ doe IL variance; r(a, ilit) genetic correlation between additive effect and litter IL, r(a, idoe) genetic correlation between additive effect and doe IL, r(ilit, idoe) genetic correlation between litter IL and doe IL

Posterior means of variance components associated with IL effects and of the corresponding genetic correlations between them and with the additive genetic variance are shown in Table 3.

Posterior means of σ²_i_ₗ_i_ₜ ranged from 44.9 to 72.2 across models, whereas those for σ^2^_idoe_ ranged from 66.4 to 118.5. Note that these variance estimates are large because they correspond to a hypothetical individual with complete inbreeding (F = 1.00). Because variance is a quadratic function of the scaling factor, rescaling to F = 0.10 requires dividing these variances by 10^2^ and by 4^2^ for F = 0.25. Thus, for example, for model 0, the estimated of σ^2^_ilit_ would be 0.72 for F = 0.10 and 4.51for F = 0.25.

Estimates of genetic correlations between the additive genetic and IL effects were generally low and showed considerable uncertainty (Table 3). For litter IL, estimates of genetic correrlation between additive effect and litter IL (r(a, i_lit_)) ranged from 0.24 to 0.39. In contrast, estimates of correlations between the additive effect and the maternal IL (r(a, i_doe_)) were negative (ranging from − 0.21 to − 0.42), although the corresponding HPD95% intervals were wide and included zero in all cases. The estimate of the correlation between the litter and maternal IL effects, r(i_lit_,i_doe_), was also negative (–0.53) for model 1, although its HPD95% was very broad (from − 0.99 to 0.50),.

Figure 1 presents posterior distributions of litter- and doe ID estimates, together with those for the associated variances of the IL for litter size based on Model 0, which was selected as the best-supported model based on DIC.

### Genetic trends

**Fig. 2:**
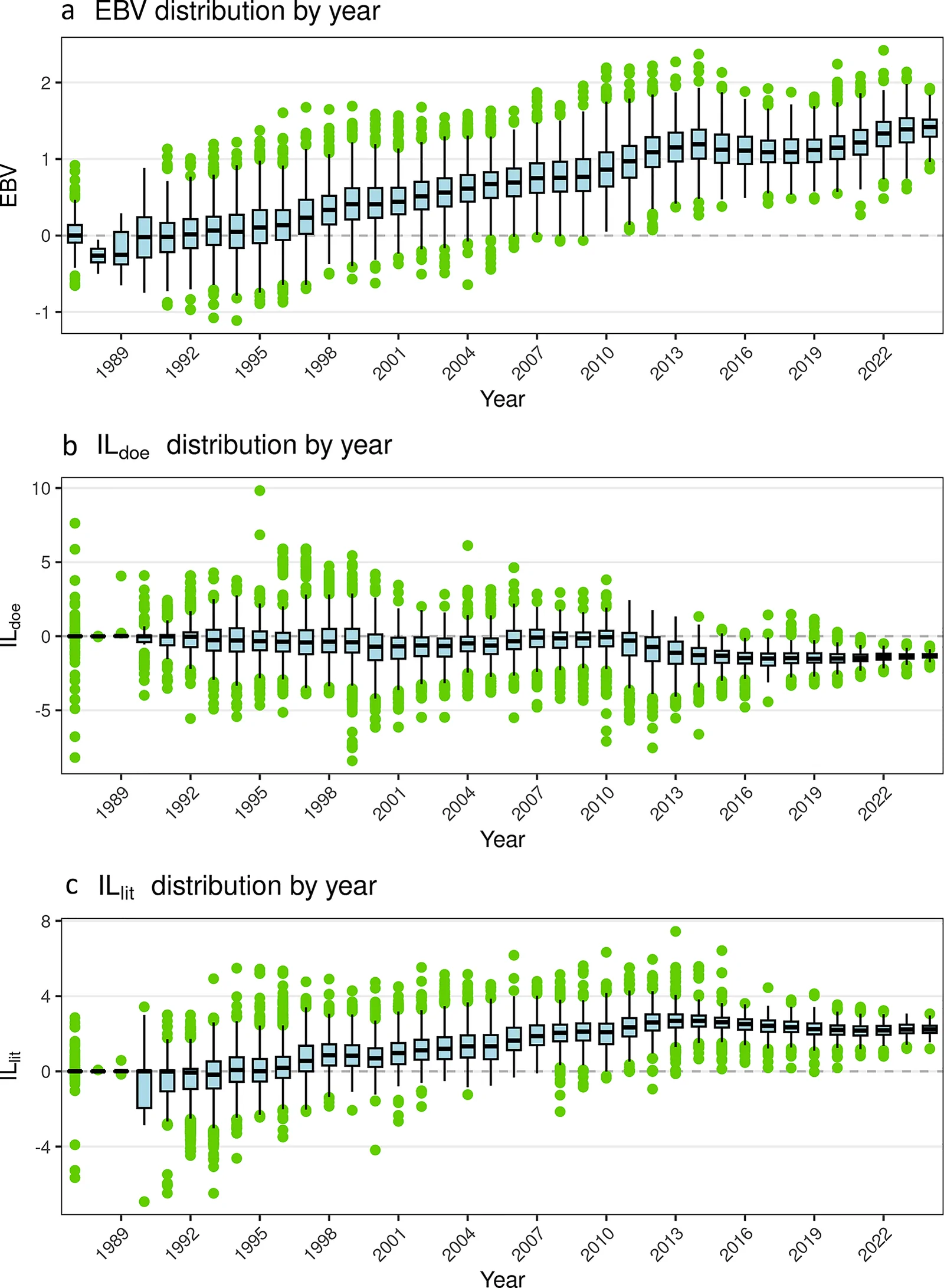
Temporal trends of genetic parameters for Number Born Alive (NBA). Annual distributions of Estimated Breeding Values (EBV) (**a**), Inbreeding Load of the doe (IL_doe_) (**b**), and Inbreeding Load of the litter (IL_lit_) (**c**) from 1987 to 2024. Box-plots represent the interquartile range (box) and median (horizontal line). Whiskers extend to the non-outlier range, while green dots correspond to outliers

Estimated breeding values (EBVs) and estimates of the different IL components allow comparison of their respective genetic trends over time. Figure 2 presents boxplots for the estimates of the additive genetic effect and of the two IL components for the years 1987 to 2024. EBVs show a clear upward trend, indicating sustained genetic progress. The median increases gradually over time, while the overall dispersion remains relatively stable. A plateau-like stabilization is observed during the 2014–2020 period, followed by a renewed increase in the most recent years. In contrast, IL_doe_ displays relatively stable median values until approximately 2010, after which a downward (more negative) trend becomes evident. Conversely, IL_lit_ exhibits a continuous positive trend across years, following a pattern broadly similar to that observed for the additive genetic effect.

## Discussion

Accurate estimation and modelling of ID has become a key component in the detection of purging, defined as the deliberated use of the interaction between inbreeding and selection in order to eliminate inbreeding depression [23], and in the design of breeding strategies that explicitly account for genetic load [24]. Approaches based on IL allow identification of unfavourable mating combinations and provide a framework for implementing artificial purging, while minimizing adverse fitness consequences [8]. In this study, we extended the conventional modelling framework by decomposing genetic load into systematic ID and individual-specific IL components, which were estimated separately at the doe and litter levels. This modelling method based on IL was for the first time applied to the number of individuals born alive (NBA), explicitly accounting for both maternal and litter-level ID effects, which are biologically plausible for this trait.

The low inbreeding coefficient for both does and litters, as well as the large number of individuals with F < 0.05 confirm the good practice of the breeding scheme, consisting of a circular mating system in which the population is divided into four groups. Based on this division, matings are arranged as follows: females from group 1 are mated with males from group 4; females from group 2 with males from group 1; females from group 3 with males from group 2; and females from group 4 with males from group 3. Offspring are then assigned to the group of their sire [25]. As reported in previous studies [11], the contribution of ancestors to the inbreeding coefficients is heterogeneous. Only a small proportion of the population (3236 individuals) contributed to the inbreeding of at least one other individual. The maximum number of inbreeding coefficients to which a single individual contributed was 57,825, whereas 498 individuals contributed to fewer than 10 coefficients.

Estimates of ID effects are consistent with previous findings in rabbits [26–29] and other species [30–32], as both maternal and litter inbreeding negatively affected NBA. However, the impact of litter inbreeding was consistently stronger than that of maternal inbreeding, being higher in 91% of the Gibbs samples. Scaled on the trait mean (NBA = 9.02 ± 3.43), model 0 estimates correspond to a reduction of − 0.57 (b_Flit_) and − 0.33% (b_Fdoe_) per 1% increase in homozygosity.

To our knowledge, this is the first formal demonstration in rabbits of a statistically supported difference between doe and litter ID; a comparable Bayesian analysis has previously been reported only in pigs [32]. The observed pattern is biologically plausible: pre- and post-implantation embryonic survival is acutely sensitive to homozygosity, since recessive deleterious alleles are directly exposed in inbred embryos [33]. The maternal component, by contrast, acts through traits of adult physiology (uterine capacity and ovulation rate) that operate in a different developmental and functional context. Confirmation of this finding in larger datasets and additional populations would be valuable.

The magnitudes of ID that we report (-0.33% for maternal ID and − 0.57% for litter ID per 1% homozygosity) are consistent with, and in some cases slightly higher than values from previous meta-analyses of reproductive traits in livestock. Doekes et al. [34] reported an average inbreeding depression of -0.30% per 1% inbreeding for reproduction-related traits across multiple species, while Leroy [3] reported − 0.18% for litter size, -0.25% for maternal litter size, -0.69% for number of offspring weaned, and − 0.46% for maternal number of offspring weaned. Our estimates therefore fall within the published range, reinforcing the well-established pattern that reproductive traits are among the most sensitive to ID in livestock.

Wider HPD95% for maternal inbreeding, which included zero in the case of model 1, suggest that the effect of maternal ID was weaker or less precisely estimated. Interestingly, although the litter effect was larger in magnitude, maternal IL accounted for a greater proportion of the variance. The wide HPD95% intervals indicate substantial uncertainty around these estimates but still suggest that maternal genetic background plays a critical role in shaping reproductive outcomes, potentially through mechanisms such as the uterine environment.

Model 0, which included both maternal and litter IL effects, provided the best fit based on DIC, supporting the importance of jointly modelling both sources of IL. This result highlights the value of the IL methodology relative to traditional regression approaches, which implicitly assume homogeneous inbreeding effects across individuals. However, the available data were insufficient to reliably estimate the genetic correlation between these components, as reflected by their broad posterior distributions that included zero and higher DIC values for models that included non-zero genetic correlations (Model 1), suggesting over-parameterization. Therefore, Model 0 was preferred because it offered a more parsimonious specification.

The observed genetic trends for additive genetic values and IL values, are consistent with the estimated directions of the genetic correlations. Although the Pannon White rabbit population has not been directly selected for NBA, long-term selection for litter weight at day 21 and average daily gain could have led to an increase of more than one kit born alive per litter. This response was accompanied by correlated genetic changes in IL, characterized by a negative trend in maternal IL (IL_doe_) and a positive trend in litter IL (IL_lit_). These patterns indicate that sustained selection on NBA alone may lead to asymmetric correlated responses in maternal and litter components of genetic load. Consequently, implementation of a weighted selection index that explicitly accounts for NBA, IL_doe_, and IL_lit_, could help mitigate undesirable changes in maternal IL, while preserving genetic progress in reproductive performance.

From an applied perspective, these results suggest that rabbit breeding programs could benefit from explicitly incorporating IL information when designing selection and mating schemes. The identification of lineages that carry elevated IL may enable breeders to prioritize matings that reduce the expression of deleterious recessive alleles without substantially limiting genetic gain. Speculatively, the negative trend observed for IL_doe_ may reflect indirect selection on maternal components of reproduction, such as uterine capacity and ovulation rate, which could favour intermediate embryo size and more efficient maternal resource allocation. Such stabilizing selection at the maternal level may reduce the expression of deleterious recessive alleles affecting maternal performance, while simultaneously allowing increased expression of litter-level IL through intensified embryo competition. Moreover, the distinct trends observed for maternal and litter IL highlight the importance of jointly considering dam and progeny contributions to reproductive performance, rather than focusing on a single component in isolation.

In line with the composite nature of NBA, as described above, the divergent patterns of the genetic trends observed for maternal and litter IL likely reflect partially distinct genetic architectures underlying each of these components.

The Pannon White population has been under sustained selection for litter weight at day 21 and average daily gain [25]. Under this selection regime, two complementary processes may contribute to the observed temporal trends in the inbreeding loads: (i) an increase in IL_lit_, consistent with indirect purging, and (ii) a decrease in IL_doe_, consistent with an unfavourable correlated response to selection. For IL_lit_, the negative effects of deleterious recessive alleles are expressed when increased homozygosity occurs in the embryo. Consequently, selection against reduced litter performance may indirectly remove deleterious recessive alleles affecting embryonic viability and pre- and post-implantation survival from the population. This indirect purging could progressively reduce the magnitude of IL_lit_, resulting in the positive temporal trend observed in Fig. 2. In contrast, IL_doe_ is associated with the inbreeding load expressed in the doe, and therefore the underlying deleterious alleles are expressed in adult does rather than directly through embryo inbreeding. Selection for litter performance may therefore generate a correlated response in IL_doe_ rather than directly purging the alleles contributing to this load. Consistent with our results, independent empirical evidence in dairy sheep [11] reported a negative genetic correlation between the additive genetic effect and the dam inbreeding load. This negative genetic correlation may reflect antagonistic pleiotropic effects, whereby alleles increasing the additive genetic merit for the selected trait are associated with less favourable effects on the maternal inbreeding load. Consequently, continued selection for the trait could result in an unfavourable correlated response in IL_doe_. Therefore, the combination of indirect purging on the litter side and an unfavourable correlated response on the doe side may explain the opposite trends between both IL in Fig. 2.

Selection for increased EBV may have indirectly reduced the IL associated with the litter component, contributing to the purging of deleterious alleles affecting embryo viability and survival. In contrast, previous evidence [11] indicates a negative relationship between doe IL and direct breeding values for NBA of the doe. Because the alleles that influence maternal traits are not necessarily the same as those that affect progeny-related traits, these components may respond differently to selection, thereby providing a plausible explanation for the contrasting trends in both IL observed.

## Conclusions

This study demonstrates the feasibility and value of jointly modelling maternal and litter inbreeding loads for the number of individuals born alive in rabbits. By decomposing genetic load across biological levels, this approach provides a more nuanced characterization of inbreeding depression than traditional regression-based methods and offers a robust framework for supporting informed selection and mating decisions. Future research should focus on validating these findings in larger datasets and across diverse populations and species, as well as on integrating genomic information to identify the genetic architecture underlying maternal and litter-specific inbreeding load components. Furthermore, models that account for the evolution of ID across generations could also be explored.

## Supplementary Information

Below is the link to the electronic supplementary material.

**Supplementary Material 1**: Additional statistical models. Additional file including two further statistical models including litter and sire direct genetic additive effect that were used to support the decision not to include the additive genetic effect of litter individuals in the complete model

**Supplementary Material 2**: Temporal Evolution of F_lit_ an F_doe_. Graphical representation of annual distributions of inbreeding coefficients for litters (F_lit_) (a) and does (F_doe_) (b). Box-plots represent the interquartile range (box) and median (horizontal line). Whiskers extend to the non-outlier range, while green dots correspond to outliers

## Figures and Tables

**Fig. A1:**
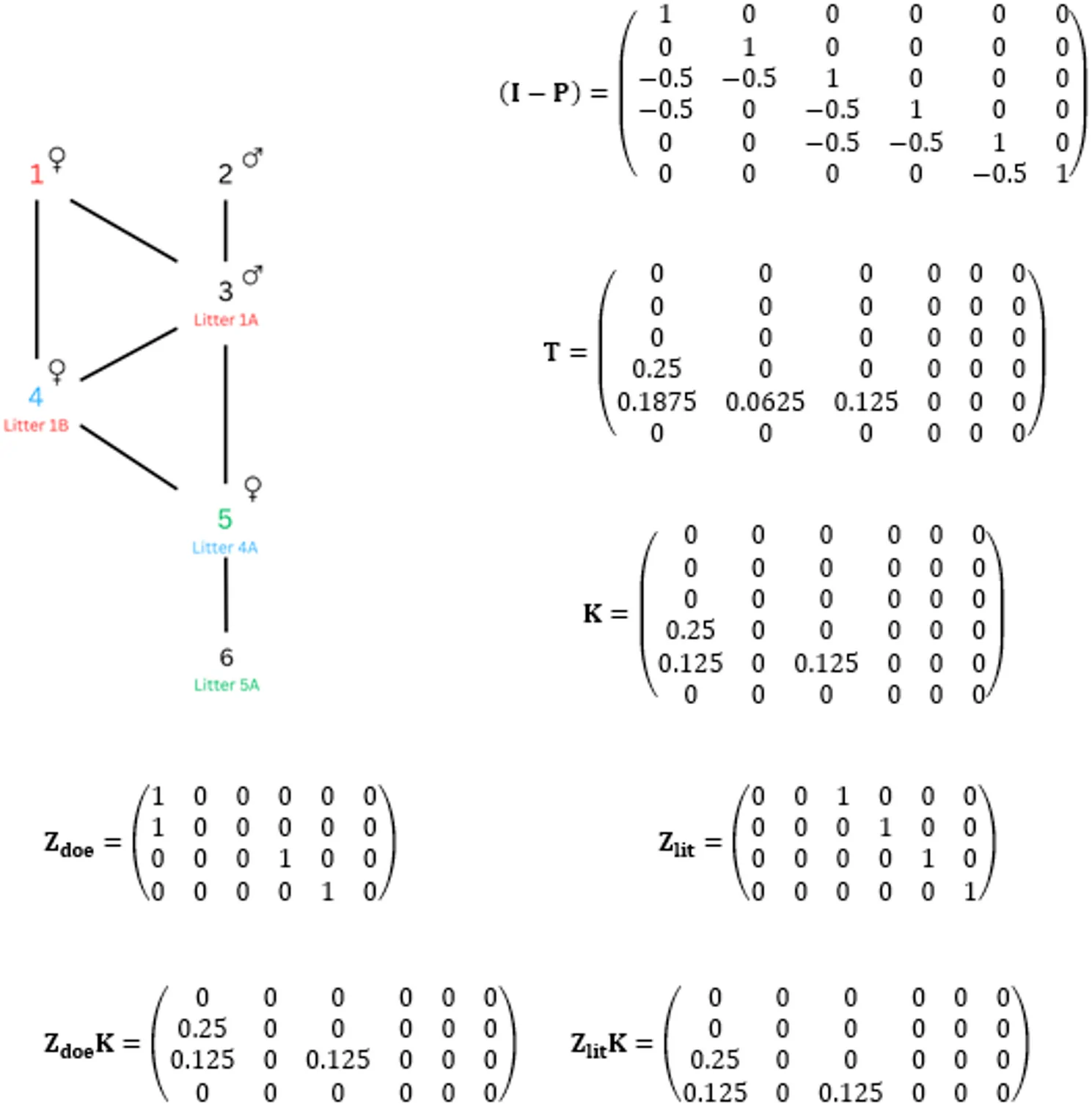
Example of the two pathways of the inbreeding load from a dummy pedigree

## Data Availability

The data analysed during the current study are not publicly available because they are owned by the Experimental Rabbit Farm of the Hungarian University of Agriculture and Life Sciences but are available from the corresponding author upon reasonable request and with permission of the owner’s representative Prof. Dr. Nagy István. The Fortran 90 program used for the analyses is available from the corresponding author upon reasonable request.

## Appendix

### Explanation of the two pathways of the inbreeding load

Appendix Fig. A1 presents a small pedigree designed to illustrate the different quantities involved in the calculation of inbreeding load (IL) when applying either the doe or the litter pathway using the correspondent incidence matrices, **Z**_**doe**_ for doe pathway and **Z**_**lit**_ for litter pathway. Colours are used to match each record (litter) with its corresponding doe, thereby facilitating interpretation.

Specifically:

- Records 1A and 1B (corresponding to individuals 3 and 4, respectively) are associated with doe 1.
- Record 4A (the litter containing individual 5) corresponds to doe 4.
- Record 5A (individual 6) corresponds to doe 5.

As described in the main text, the (**I** − **P**) matrix is a 6 × 6 matrix (number of individuals × number of individuals), with ones on the diagonal and − 0.5 in the positions linking each individual to its parents. This example highlights how the use of the Mendelian decomposition differs between pathways (**Z**_**doe**_**K** and **Z**_**lit**_**K**), even though the same pedigree information and individuals are considered. The key distinction lies in how the individual to which the decomposition is taken:

- Under the doe pathway (**Z**_**doe**_**K**), the decomposition is applied to the doe associated with each litter.
- Under the litter pathway (**Z**_**lit**_**K**), the decomposition is applied to the individuals born within the litter.

For example, record 5 A exhibits a high level of inbreeding when the doe pathway is used. In contrast, under the litter pathway, this record does not contribute to inbreeding, because the offspring resulting from individual 5 mated to an unknown sire produces a descendant with no inbreeding coefficient. After computing **Z**_**doe**_**K** and **Z**_**lit**_**K** matrices, clear structural differences emerge between them, resulting in distinct numerical values. These differences must be accounted for in the mixed model equations, as they directly affect the estimation of inbreeding load.
