# Supplementary material for: Quantifying genetic load through joint modelling of inbreeding depression and inbreeding load for litter size in rabbits
Source: Genet Sel Evol. 2026 Sep 20;58:69. doi: 10.1186/s12711-026-01090-5 (PMC13589861; doi:10.1186/s12711-026-01090-5)
Supplement: Supplementary file 1 [file 12711_2026_1090_MOESM1_ESM.docx]

**Supplementary File 1.** Additional models

This additional file presents two further statistical models that were used to support the decision not to include the additive genetic effect of litter individuals in the complete model.

**y = f_doe_*****b_fdoe_*+ f_lit_*****b_flit_* + Xd + Sh + Wp + Z_doe_a_doe_ + Z_sire_a_sire_ + e (S1)**

**y = f_doe_*b_fdoe_*+ f_lit_*b_flit_* + Xd + Sh + Wp + Xd + Z_doe_a_doe_ + Z_lit_a_lit_ + e (S2)**

In this case, as in the models of the main manuscript, 𝐲 is the vector of phenotypic records (NBA), ***b_fdoe_*** and ***b_flit_*** are the regression coefficients representing the inbreeding depression parameters per unit of inbreeding, **f_doe_** and **f_lit_** are the vectors of the total inbreeding coefficients of the doe and the individuals of the litter, and represents the average ID of does and litters, respectively, ***d*** is the vector of fixed effects (including order of parity with 6 levels), ***h*** is the vector of random herd–year–season (318 levels), ***p*** is the vector of the permanent environmental maternal effect and **a_doe_** is the vector of the direct (doe) additive genetic effects. Now it is included **a_sire_** in model S1 and **a_lit_** in model S2 that accounted for the vector of the direct additive effect at the sire level and direct additive effect at the litter level respectively. Further, **X, S, W, Z_doe_, Z_sire_** and **Z_lit_** are the incidence matrices. We note that Model S2 is only an approximation, since representing the litter through a single equivalent individual is not theoretically correct; we used it solely to gauge the potential magnitude of direct genetic effects at the litter level. At table S1, we can observe the result of the variance components when using these models.

**Table S1.** Posterior mean with HPD95% of shared variance components and heritabilities.

|  | MODEL | |
| --- | --- | --- |
|  | S1 | S2 |
| σ^2^_a_doe_ [HPD95%] | 0.55 [0.39, 0.71] | 0.50 [0.33, 0.66] |
| σ^2^_a_sire_ [HPD95%] | 0.04 [0.01, 0.07] | -- |
| σ^2^_a_lit_ [HPD95%] | -- | 0.17 [0.07, 0.28] |
| σ^2^_h_ [HPD95%] | 0.25 [0.18, 0.31] | 0.25 [0.18, 0.31] |
| σ^2^_p_ [HPD95%] | 0.99 [0.82, 1.16] | 0.99 [0.83, 1.16] |
| σ^2^_e_ [HPD95%] | 9.78 [9.61,9.95] | 9.69 [9.51,9.88] |
| h^2^_doe_ [HPD95%] | 0.047 [0.033, 0.061] | 0.043 [0.028, 0.057] |
| h^2^_sire_ [HPD95%] | 0.003 [0.001, 0.006] | -- |
| h^2^_li t_[HPD95%] | -- | 0.015 [0.006, 0.024] |

σ^2^_a_doe_ additive variance (maternal); σ^2^_a_sire_ additive variance (paternal); σ^2^_a_lit_ additive variance (litter); σ^2^_h_ random herd-year-season variance; σ^2^_p_ permanent variance; σ^2^_e_ residual variance; h^2^_doe_	maternal heritability_;_ h^2^_sire_	paternal heritability; h^2^_lit_ litter heritability.

Both alternative specifications consistently show that direct additive genetic effects at the litter level account for only a negligible share of the phenotypic variance in number born alive (paternal heritability ≈ 0.003 in Model S1; litter heritability ≈ 0.015 in Model S2). Based on these results, and in line with the established literature on litter size in rabbits and pigs, we conclude that a model including only the maternal additive genetic effect adequately captures the relevant additive variation for this trait. Consequently, the direct additive effect at the litter level can be omitted from the final model without compromising its explanatory power.
